# Supplementary material for: OXR1 maintains the retromer to delay brain aging under dietary restriction
Source: Nat Commun. 2024 Jan 11;15:467. doi: 10.1038/s41467-023-44343-3 (PMC10784588; doi:10.1038/s41467-023-44343-3)
Supplement: Supplementary file 1 — Supplementary Information [file 41467_2023_44343_MOESM1_ESM.pdf]

OXR1 maintains the retromer to delay brain aging under dietary restriction

Supplementary Information

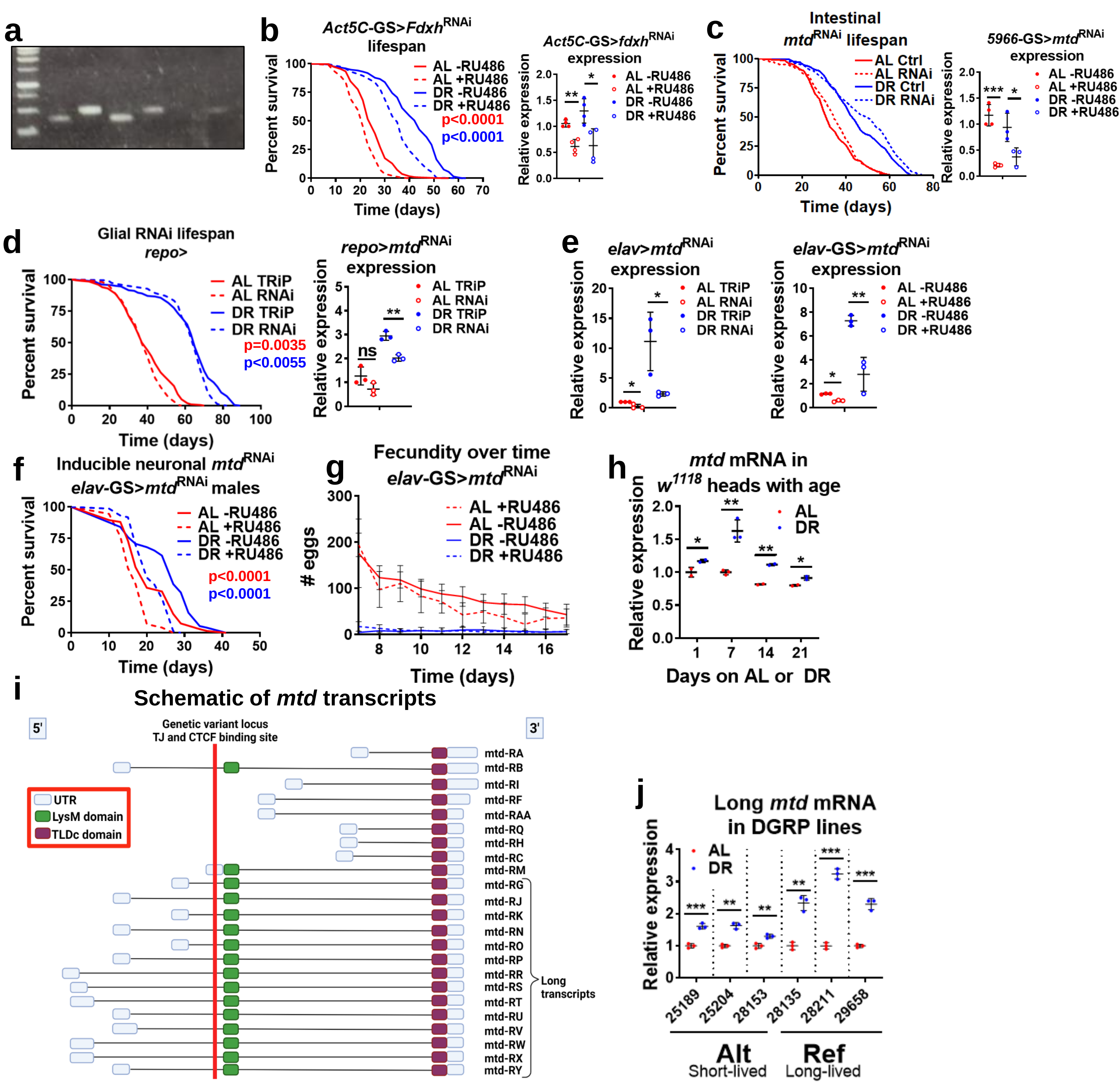

k

mtd allele binding candidates				
Transcription Factor	Binding site	Signal value	q value	Peak
CTCF	3R:5298551 - 3R:5299881	24335.0	4.012	648
TJ	3R:5298549 - 3R:5299120	1636.2	4.371	250

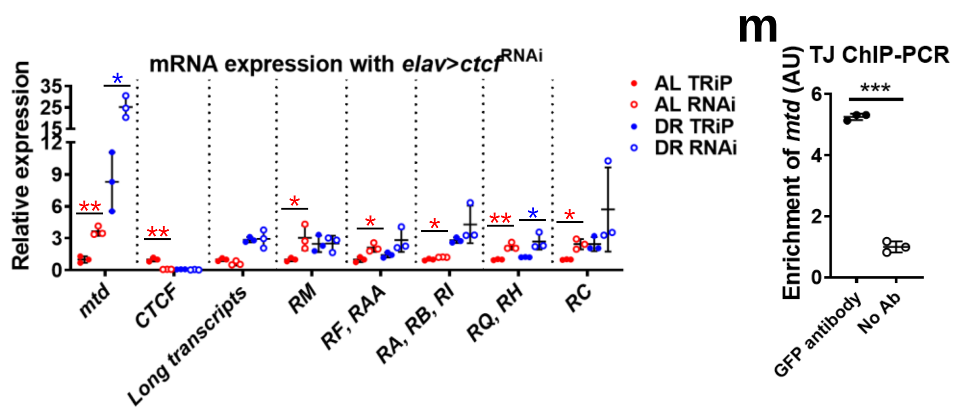

Supplementary Fig. 1: GWAS candidate gene validation and genetic regulators of *mtd*.

a, Shift in DNA band size for *mtd* variant region in DGRP lines. DGRP strains from left to right are BL29655, 28243, 28211, 25204, 25201, 25189. Higher bands have the short-lived allele. **b**, Adulthood-specific RU486-induced whole body RNAi for *Ferredoxin* (dashed lines) reduces lifespan under both dietary conditions. AL in red, DR in blue. qRT-PCR for *Fdxh* expression on right. **c**, Adulthood-specific RU486-induced intestinal *mtd*^{RNAi} (dashed lines) does not reduce lifespan. qRT-PCR for *mtd* expression on right. **d**, Constitutively active glial *mtd*^{RNAi} does not reduce lifespan. qRT-PCR results on right. **e**, Validation of reduced *mtd* expression in flies with constitutively active neuronal *mtd*^{RNAi} (left) or RU486-inducible neuronal *mtd*^{RNAi} (right). AL in red, DR in blue. Solid circles = controls, open circles = RNAi. Values normalized to AL mean value. **f**, RU486-inducible neuronal *mtd*^{RNAi} in adulthood shortens lifespan in males independent of diet. **g**, RU486-induced neuronal *mtd*^{RNAi} does not impact number of eggs laid. **h**, Expression of *mtd* in heads of w^{1118} flies increases by DR and declines with age. Values normalized to AL value on day 1. **i**, Schematic of *mtd* gene region. Grey boxes = untranslated regions, green boxes = LysM domain coding region, purple boxes = TLDc domain coding region. Red line = site of genetic variation across the DGRP lines and site of TJ- and CTCF-binding affinities. **j**, Expression of *mtd* long transcripts (defined in **i**) in the heads of six DGRP strains either possessing the short-lived (left) or long-lived (right) allele are significantly elevated after 7 days of DR (blue). **k**, CTCF and TJ significantly bind to *mtd* variant site. **l**, Total *mtd* mRNA levels and shorter *mtd* transcripts in the heads of flies are upregulated with constitutively active neuronal *ctcf*^{RNAi}. Values normalized to AL control levels. Red = AL, blue = DR. Solid circles = controls, open circles = with *ctcf*^{RNAi}. **m**, ChIP-PCR pulldown of GFP-tagged TJ at *mtd* variant site. p values in **b-d** and **f** determined by log-rank test. p values in other panels determined by two-sided t-test. For all panels, * = $p<0.05$, ** = $p<0.005$, *** = $p<0.0005$ and error bars represent mean values +/- SD. n = 5 bodies (**b-c**) or 50 heads (**d-e, h, j, l-m**) across minimum 3 biological replicates. For **g**, n = 8 breeding chambers of 5 females and 3 males. Figure S1i was generated using BioRender (publishing license: BN266MCVSU)

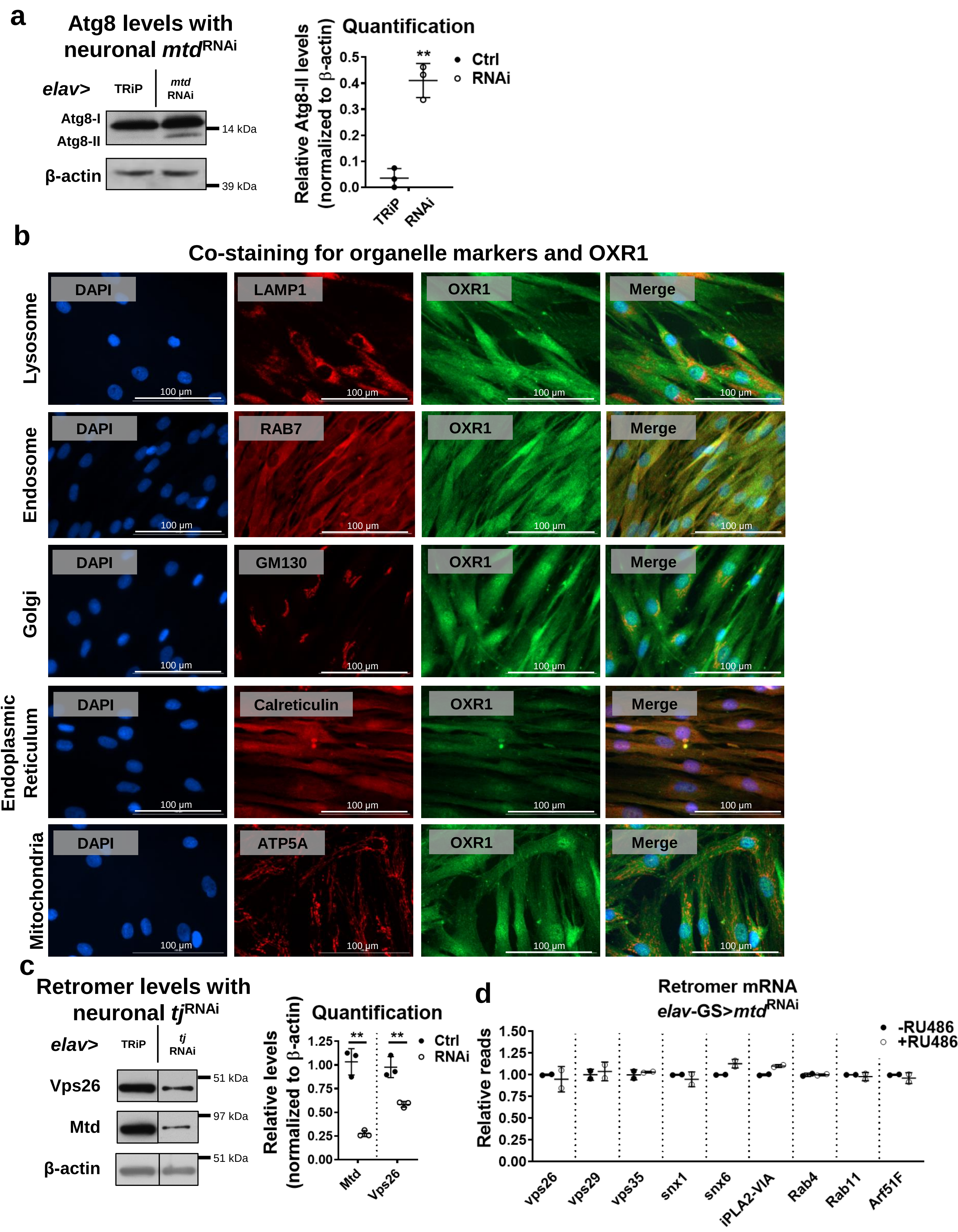
a
Atg8 levels with neuronal mtdRNAi
elav>
TRiP
mtd RNAi
Atg8-I
Atg8-II
14 kDa
β-actin
39 kDa
Quantification
Relative Atg8-II levels (normalized to β-actin)
**
Ctrl
RNAi
TRiP
RNAi
b
Co-staining for organelle markers and OXR1
Lysosome
Endosome
Golgi
Endoplasmic Reticulum
Mitochondria
DAPI
LAMP1
RAB7
GM130
Calreticulin
ATP5A
OXR1
Merge
100 μm
c
Retromer levels with neuronal tjRNAi
elav>
TRiP
tj RNAi
Vps26
Mtd
β-actin
51 kDa
97 kDa
51 kDa
Quantification
Relative levels (normalized to β-actin)
Ctrl
RNAi
Mtd
Vps26
d
Retromer mRNA
elav-GS>mtdRNAi
-RU486
+RU486
Relative reads
vps26
vps29
vps35
snx1
snx6
iPLA2-VIA
Rab4
Rab11
Arf51F

Supplementary Fig. 2: OXR1 localizes to the endosome and transcriptional changes in retromer expression are not the cause of OXR1-related lysosome dysfunction.

a, Atg8-II levels are elevated in heads of flies with constitutively active pan-neuronal *mtd*^{RNAi}. Quantified on right. n = 50 fly heads for 3 independent experiments. **b**, Immunocytochemistry of OXR1 (green) counterstained with various organelle markers (red) shows co-localization to the endosome. Green = LAMP1 for lysosome, GM130 for Golgi, Calreticulin for endoplasmic reticulum, ATP5A for mitochondria, and RAB7 for endosome. Mitochondria were analyzed due to previously identified roles of OXR1 in oxidative stress response. Representative images were chosen from 10 images across 3 independent experiments. **c**, Vps26 and Mtd levels are reduced in heads of flies with constitutively active pan-neuronal *tj*^{RNAi}. n = 50 fly heads for 3 independent experiments. **d**, mRNA transcript reads for retromer-related genes from RNA-seq dataset show no significant changes with *mtd*^{RNAi}. For **a** and **b-c**, error bars represent mean value across replicates +/- SD. For all panels, * = $p<0.05$, ** = $p<0.005$, *** = $p<0.0005$.

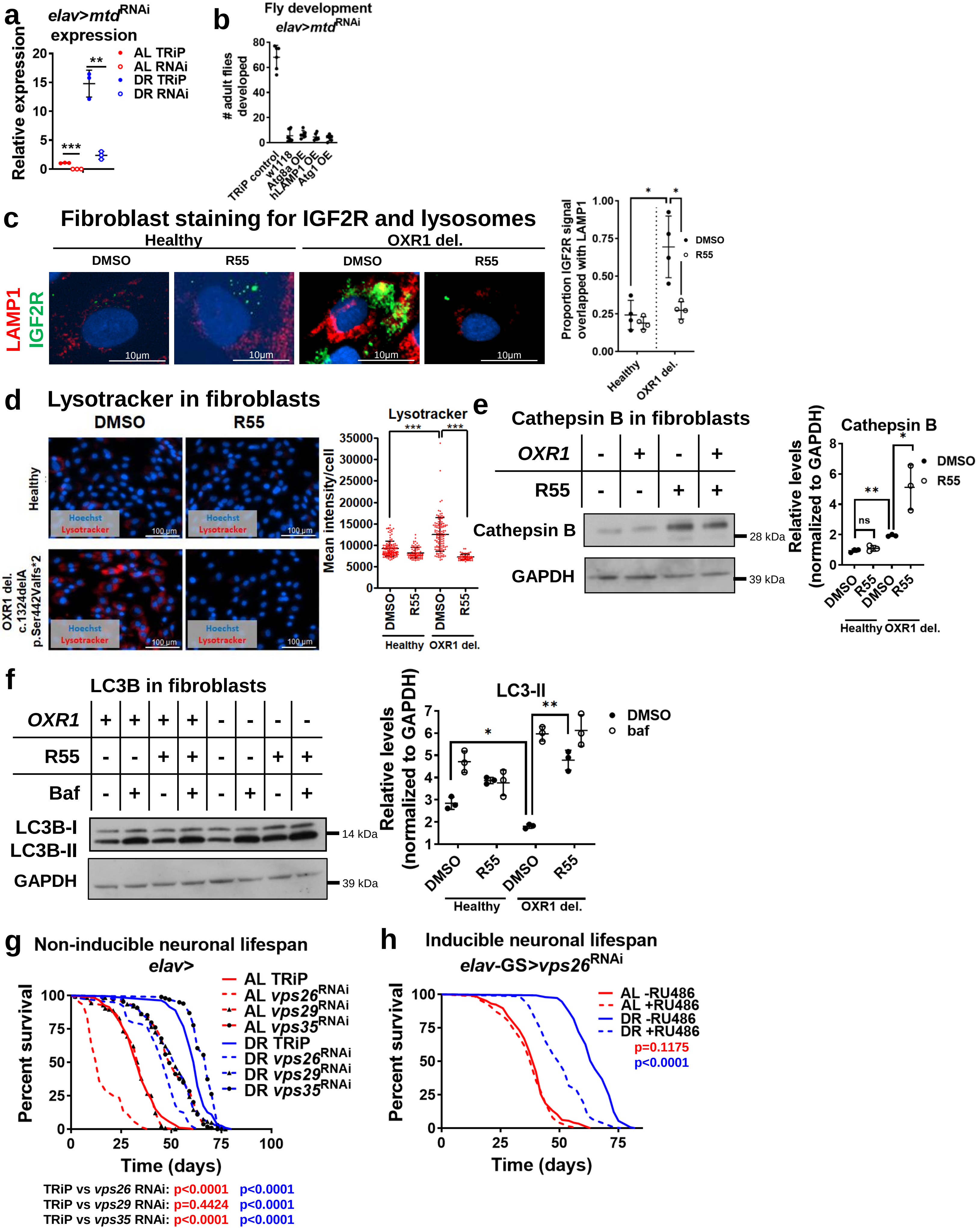

a
elav>mtd RNAi expression
Relative expression
AL TRiP
AL RNAi
DR TRiP
DR RNAi
b
Fly development
elav>mtd RNAi
adult flies developed
TRiP control
w1118
Atg8a OE
hLAMP1 OE
Atg1 OE
c
Fibroblast staining for IGF2R and lysosomes
Healthy
OXR1 del.
DMSO
R55
LAMP1
IGF2R
10µm
Proportion IGF2R signal overlapped with LAMP1
d
Lysotracker in fibroblasts
Healthy
OXR1 del. c.1324delA p.Ser442Valfs*2
Hoechst
Lysotracker
100 µm
Lysotracker
Mean intensity/cell
e
Cathepsin B in fibroblasts
OXR1
R55
Cathepsin B
GAPDH
28 kDa
39 kDa
Relative levels (normalized to GAPDH)
ns
f
LC3B in fibroblasts
Baf
LC3B-I
LC3B-II
14 kDa
LC3-II
baf
g
Non-inducible neuronal lifespan
elav>
AL TRiP
AL vps26 RNAi
AL vps29 RNAi
AL vps35 RNAi
DR TRiP
DR vps26 RNAi
DR vps29 RNAi
DR vps35 RNAi
Percent survival
Time (days)
TRiP vs vps26 RNAi: p<0.0001 p<0.0001
TRiP vs vps29 RNAi: p=0.4424 p<0.0001
TRiP vs vps35 RNAi: p<0.0001 p<0.0001
h
Inducible neuronal lifespan
elav-GS>vps26 RNAi
AL -RU486
AL +RU486
DR -RU486
DR +RU486
p=0.1175
p<0.0001

Supplementary Fig. 3: R55 rescues lysosomal phenotypes associated with *mtd* and OXR1 deficiency.

a, Validation of *mtd* expression in constitutively active *mtd*^{RNAi} used in retromer rescue experiments. n = 50 fly heads across 3 independent experiments. **b**, Developmental defects in flies with constitutively active neuronal *mtd*^{RNAi} is not rescued by overexpression of autophagy genes *Atg8*, *Lamp1*, *p62*, or *Atg1*. n = 6 breeding chambers of 5 females with 3 males. **c**, Co-localization of IGF2R with lysosomal marker LAMP1 is upregulated in human fibroblasts with a loss-of-function *OXR1* mutation, which is rescued by 10 µM R55. Blue = DAPI, green = IGF2R, red = LAMP1. Quantification on right. n = 50,000 cells across 4 biological replicates per condition. **d**, Lysotracker staining is elevated in live cell images of fibroblasts with homozygous *OXR1* mutation. Nucleus stained with Hoechst (blue), lysosomes stained with Lysotracker (red), which is rescued by treatment with 10 µM R55. Lysotracker intensity per cell quantified beside images. n = 10 field images cells across 4 independent experiments. **e**, Cathepsin B levels are elevated in mutant fibroblasts, and further elevated in cells treated with 10 µM R55. n = lysates from 500,000 cells across 3 independent experiments. **f**, Autophagic flux is elevated in cells with *OXR1* mutation, which is lowered by 10 µM R55 supplementation. n = lysates from 500,000 cells across 3 independent experiments. **g**, Constitutively active neuronal RNAi for *vps26* reduces lifespan under both diets, RNAi for *vps29* reduces lifespan under DR conditions. **h**, RU486-inducible neuronal RNAi for *vps26* reduces lifespan only under DR conditions. Across all panels, error bars represent mean values +/- SD. For **a-f**, p value determined by two-way t-test. For **g-h**, p value determined by log-rank test. For all panels, * = $p<0.05$, ** = $p<0.005$, *** = $p<0.0005$.

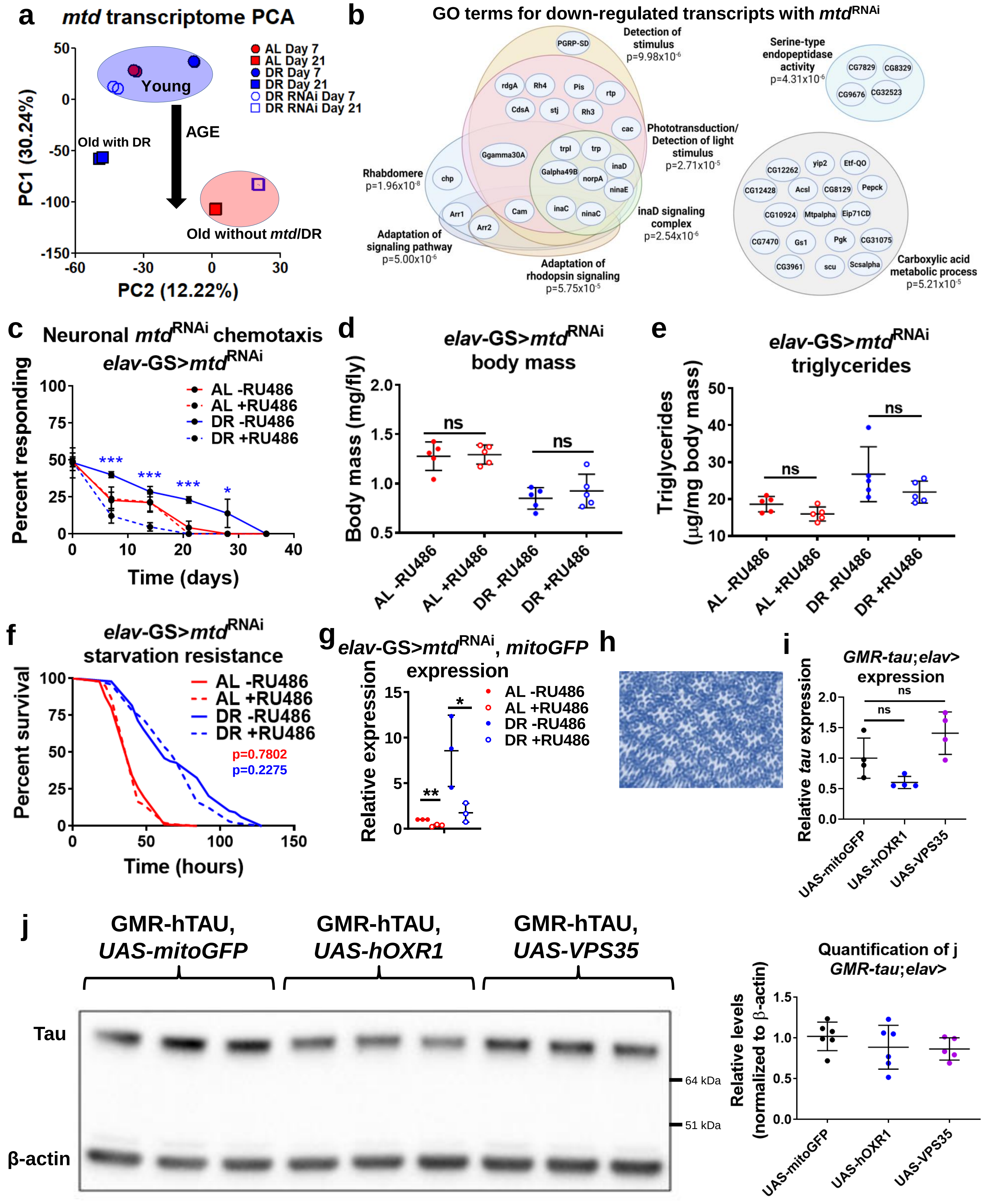
a
mtd transcriptome PCA
Young
Old with DR
AGE
Old without mtd/DR
AL Day 7
AL Day 21
DR Day 7
DR Day 21
DR RNAi Day 7
DR RNAi Day 21
PC1 (30.24%)
PC2 (12.22%)
b
GO terms for down-regulated transcripts with mtdRNAi
Detection of stimulus p=9.98x10-6
Phototransduction/ Detection of light stimulus p=2.71x10-5
inaD signaling complex p=2.54x10-6
Adaptation of rhodopsin signaling p=5.75x10-5
Adaptation of signaling pathway p=5.00x10-6
Rhabdomere p=1.96x10-8
Serine-type endopeptidase activity p=4.31x10-6
Carboxylic acid metabolic process p=5.21x10-5
c
Neuronal mtdRNAi chemotaxis
elav-GS>mtdRNAi
Percent responding
Time (days)
AL -RU486
AL +RU486
DR -RU486
DR +RU486
d
elav-GS>mtdRNAi body mass
Body mass (mg/fly)
e
elav-GS>mtdRNAi triglycerides
Triglycerides (µg/mg body mass)
f
elav-GS>mtdRNAi starvation resistance
Percent survival
Time (hours)
p=0.7802
p=0.2275
g
elav-GS>mtdRNAi, mitoGFP expression
Relative expression
h
i
GMR-tau;elav> expression
Relative tau expression
UAS-mitoGFP
UAS-hOXR1
UAS-VPS35
j
GMR-hTAU, UAS-mitoGFP
GMR-hTAU, UAS-hOXR1
GMR-hTAU, UAS-VPS35
Tau
β-actin
64 kDa
51 kDa
Quantification of j
GMR-tau;elav>
Relative levels (normalized to β-actin)

Supplementary Fig. 4: Neuronal loss of *mtd* induces sensory transcriptomic changes and not metabolic dysregulation, and rescues phenotypes in a fly model of tauopathy.

a, PCA from RNA-seq data show that heads from old flies under AL segregate with heads from flies under DR with RU486-inducible neuronal *mtd*^{RNAi}. **b**, GO terms from RNA-seq analysis show alterations in genes relating to vision and stimulus detection. **c**, Flies undergoing DR with RU486-induced pan-neuronal *mtd*^{RNAi} have reduced responsiveness to chemical attractant 1-hexanol. n = minimum 50 flies across 5 independent experiments. **d**, Body mass is not affected by RU486-induced pan-neuronal *mtd*^{RNAi} after 7 days on AL (red) or DR (blue) diet. n = 3 flies for 5 independent experiments. **e**, Triglyceride levels are not affected by RU486-induced pan-neuronal *mtd*^{RNAi} after 7 days on an AL (red) or DR (blue) diet, normalized to body mass. n = 3 flies for 5 independent experiments. **f**, Starvation resistance is not affected by RU486-induced pan-neuronal *mtd*^{RNAi} after 7 days on an AL (red) or DR (blue) diet. **g**, Validation of *mtd* expression in RU486-inducible *mtd*^{RNAi} used in TUNEL staining. n = 50 fly heads for 3 independent experiments. n = minimum 100 flies per condition for 3 independent experiments. **h**, Fly photoreceptors are not altered by GMR-promoted expression of mutant *Tau*. n = eyes of 20 flies per condition for 4 independent experiments. **i**, Validation of *tau* mRNA expression in constitutive GMR-tau crosses. **j**, Western blot validation of *GMR*-driven tau overexpression in flies with and without neuronal overexpression of *hOXR1* or *VPS35*. n = 25 heads per sample for 3 biological replicates. For all panels, * = $p<0.05$, ** = $p<0.005$, *** = $p<0.0005$ and error bars represent mean values +/- SD. For **c-e** and g-**j**, p value calculated by two-sided t-test. For **f**, p value calculated by log-rank test. Figure S4b was generated using BioRender (publishing license: KK266MD2Q0)
