# Supplementary material for: OXR1 maintains the retromer to delay brain aging under dietary restriction
Source: Nat Commun. 2024 Jan 11;15:467. doi: 10.1038/s41467-023-44343-3 (PMC10784588; doi:10.1038/s41467-023-44343-3)
Supplement: Supplementary file 4 — Reporting Summary [file 41467_2023_44343_MOESM4_ESM.pdf]

nature portfolio

Corresponding author(s): Pankaj Kapahi, Lisa Ellerby

Last updated by author(s): Dec 1, 2023

Reporting Summary

Nature Portfolio wishes to improve the reproducibility of the work that we publish. This form provides structure for consistency and transparency in reporting. For further information on Nature Portfolio policies, see our Editorial Policies and the Editorial Policy Checklist.

Statistics

For all statistical analyses, confirm that the following items are present in the figure legend, table legend, main text, or Methods section.

n/a	Confirmed	
☐	☒	The exact sample size (*n*) for each experimental group/condition, given as a discrete number and unit of measurement
☐	☒	A statement on whether measurements were taken from distinct samples or whether the same sample was measured repeatedly
☐	☒	The statistical test(s) used AND whether they are one- or two-sided<br>*Only common tests should be described solely by name; describe more complex techniques in the Methods section.*
☐	☒	A description of all covariates tested
☐	☒	A description of any assumptions or corrections, such as tests of normality and adjustment for multiple comparisons
☐	☒	A full description of the statistical parameters including central tendency (e.g. means) or other basic estimates (e.g. regression coefficient) AND variation (e.g. standard deviation) or associated estimates of uncertainty (e.g. confidence intervals)
☐	☒	For null hypothesis testing, the test statistic (e.g. *F*, *t*, *r*) with confidence intervals, effect sizes, degrees of freedom and *P* value noted<br>*Give P values as exact values whenever suitable.*
☒	☐	For Bayesian analysis, information on the choice of priors and Markov chain Monte Carlo settings
☒	☐	For hierarchical and complex designs, identification of the appropriate level for tests and full reporting of outcomes
☒	☐	Estimates of effect sizes (e.g. Cohen's *d*, Pearson's *r*), indicating how they were calculated

Our web collection on statistics for biologists contains articles on many of the points above.

Software and code

Policy information about availability of computer code

Data collection	Cell images were collected using BioTek Cytation 5 imaging reader. Tissue samples were analyzed using Zen Blue 3.3. Gel and membrane images were analyzed used BioRad Image Lab 2.0. qPCR data were collected using Roche Real Time Cycler 480.
Data analysis	Cell images were analyzed using Gen5 Image Prime 3.11. Tissue samples were analyzed using Zen Blue 3.3 and ImageJ. Data plotting and statistical analyses were performed using GraphPad Prism 4.

For manuscripts utilizing custom algorithms or software that are central to the research but not yet described in published literature, software must be made available to editors and reviewers. We strongly encourage code deposition in a community repository (e.g. GitHub). See the Nature Portfolio guidelines for submitting code & software for further information.

Data

Policy information about availability of data

All manuscripts must include a data availability statement. This statement should provide the following information, where applicable:

- Accession codes, unique identifiers, or web links for publicly available datasets
- A description of any restrictions on data availability
- For clinical datasets or third party data, please ensure that the statement adheres to our policy

All normalized sequencing data and curated proteomics data are available as supplemental data tables or have original sources referenced. Fly proteomics data are available from the following

link and login information:
https://massive.ucsd.edu/ProteoSAFe/private-dataset.jsp?task=d8f8a278868d42398c9ba8c772395c7b
Username: MSV000088897_reviewer
Password: winter

The raw RNA sequencing data fastq files were lost during a recent server crash. This information was previously relayed to the handling editor.

Research involving human participants, their data, or biological material

Policy information about studies with human participants or human data. See also policy information about sex, gender (identity/presentation), and sexual orientation and race, ethnicity and racism.

Reporting on sex and gender	N/A
Reporting on race, ethnicity, or other socially relevant groupings	N/A
Population characteristics	N/A
Recruitment	N/A
Ethics oversight	N/A

Note that full information on the approval of the study protocol must also be provided in the manuscript.

Field-specific reporting

Please select the one below that is the best fit for your research. If you are not sure, read the appropriate sections before making your selection.

☒ Life sciences ☐ Behavioural & social sciences ☐ Ecological, evolutionary & environmental sciences

For a reference copy of the document with all sections, see nature.com/documents/nr-reporting-summary-flat.pdf

Life sciences study design

All studies must disclose on these points even when the disclosure is negative.

Sample size	Sample sizes were selected based on previously published standards for sizes using the described models. mtd RNAi fly lifespans were performed with smaller sample sizes due to larval lethality, but were repeated multiple times to ensure replication.
Data exclusions	No data were excluded from analyses.
Replication	Experiments were replicated a minimum of 3 times except where specifically stated, including multiple replicates by different researchers. Replication was consistent across repeated experiments.
Randomization	For experiments with treatments, cells and flies were separated randomly into their specific conditions (dietary, genetic induction, or treatment group). Cells and flies were sorted into their respective assay conditions randomly for each condition.
Blinding	Blinding was not possible in this study. It is not possible to be blinded from diet-related analyses as the color of the diet is different between conditions.

Reporting for specific materials, systems and methods

We require information from authors about some types of materials, experimental systems and methods used in many studies. Here, indicate whether each material, system or method listed is relevant to your study. If you are not sure if a list item applies to your research, read the appropriate section before selecting a response.

Materials & experimental systems

n/a	Involved in the study	
☐	☒	Antibodies
☐	☒	Eukaryotic cell lines
☒	☐	Palaeontology and archaeology
☐	☒	Animals and other organisms
☒	☐	Clinical data
☒	☐	Dual use research of concern
☒	☐	Plants

Methods

n/a	Involved in the study	
☒	☐	ChIP-seq
☒	☐	Flow cytometry
☒	☐	MRI-based neuroimaging

Antibodies

Antibodies used

Rabbit polyclonal anti-OXR1 Invitrogen Cat#PA5-72405
Rabbit polyclonal anti-OXR1 abcam Cat#ab103042
Goat polyclonal anti-VPS35 abcam Cat#ab10099
Rabbit polyclonal anti-VPS26A Proteintech Cat#12804-1-AP
Rabbit polyclonal anti-VPS26B Proteintech Cat#15915-1-AP
Rabbit polyclonal anti-VPS29 Abcam Cat#ab236796
Mouse monoclonal anti-β-Galactosidase (LacZ) Promega Cat#Z3781
Rabbit polyclonal anti-LC3B Novus Biologicals Cat#NB100-2220
Rabbit polyclonal anti-Atg8 Sigma-Aldrich Cat#ABC974
Mouse monoclonal anti-Cathepsin B [CA10] abcam Cat#ab58802
Rabbit polyclonal anti-GAPDH abcam Cat#ab9485
Rabbit polyclonal anti-β-actin Cell Signaling Cat#4967
Mouse monocolonal anti-α-Tubulin [DM1A] Sigma-Aldrich Cat#T6199
Mouse monoclonal anti-Rab7 [EPR7589] abcam Cat#ab50533
Mouse monoclonal anti-elav DSHB Cat#Elav-9F8A9
Mouse monoclonal anti-EEA1 [G-4] Santa Cruz Biotechnology Cat#sc-137130
Mouse monoclonal anti-ATP5A [15H4C4] abcam Cat#ab14748
Mouse monoclonal anti-LAMP1 [H4A3] abcam Cat#ab25630
Mouse monoclonal anti-Calreticulin [FMC 75] abcam Cat#ab22683
Mouse polyclonal anti-GM130 abcam Cat#ab169276
Mouse monoclonal anti-GFP [B-2] Santa Cruz Biotech Cat#sc-9996
Mouse monoclonal anti-Tau [HT7] Invitrogen Cat#MN1000
Sheep anti-Mouse IgG HRP-linked secondary Sigma Aldrich Cat#NXA931V
Donkey anti-Rabbit IgG HRP-linked secondary Sigma Aldrich Cat#NA934V
Mouse anti-Goat IgG HRP-conjugated secondary Santa Cruz Biotech Cat#sc-2354
Alexa Fluor 488 donkey anti-rabbit IgG (H+L) secondary Invitrogen Cat#A21206
Alexa Fluor 647 donkey anti-goat IgG (H+L) secondary Invitrogen Cat#A21447
Alexa Fluor 647 donkey anti-mouse IgG (H+L) secondary Invitrogen Cat#A21238

Validation

Rabbit polyclonal anti-OXR1 Invitrogen Cat#PA5-72405 WB concentration: 1:500 Validated by RNAi in manuscript.
Rabbit polyclonal anti-OXR1 abcam Cat#ab103042 ICC concentration: 1:100 Validated against human OXR1 mutation in manuscript.
Goat polyclonal anti-VPS35 abcam Cat#ab10099 WB concentration: 1:500
ICC concentration: 1:100 Validated by KO (abcam).
Rabbit polyclonal anti-VPS26A Proteintech Cat#12804-1-AP WB concentration: 1:500 Previously validated (Hou et al., referenced in manuscript).
Rabbit polyclonal anti-VPS26B Proteintech Cat#15915-1-AP WB concentration: 1:500 Validated by molecular weight in manuscript.
Rabbit polyclonal anti-VPS29 Abcam Cat#ab236796 WB concentration: 1:500 Validated by KO (abcam).
Mouse monoclonal anti-β-Galactosidase (LacZ) Promega Cat#Z3781 WB concentration: 1:500
IHC concentration: 1:100 Validated by molecular weight in manuscript.
Rabbit polyclonal anti-LC3B Novus Biologicals Cat#NB100-2220 WB concentration: 1:150 Validated by KO (Novus Biologicals).
Rabbit polyclonal anti-Atg8 Sigma-Aldrich Cat#ABC974 WB concentration: 1:500 Validated by molecular weight in manuscript.
Mouse monoclonal anti-Cathepsin B [CA10] abcam Cat#ab58802 WB concentration: 1:500 Validated by molecular weight in manuscript.
Rabbit polyclonal anti-GAPDH abcam Cat#ab9485 WB concentration: 1:500 Validated by molecular weight in manuscript.
Rabbit polyclonal anti-β-actin Cell Signaling Cat#4967 WB concentration: 1:500 Validated by company (Cell Signaling).
Mouse monocolonal anti-α-Tubulin [DM1A] Sigma-Aldrich Cat#T6199 WB concentration: 1:500 Validated by company (Sigma-Aldrich).
Mouse monoclonal anti-Rab7 [EPR7589] abcam Cat#ab50533 WB concentration: 1:500
ICC concentration: 1:100 Validated by company (abcam).
Mouse monoclonal anti-elav DSHB Cat#Elav-9F8A9 IHC concentration: 1:100 Validated by neuronal specificity in manuscript
Mouse monoclonal anti-EEA1 [G-4] Santa Cruz Biotechnology Cat#sc-137130 ICC concentration: 1:100 Validated by company (Santa Cruz Biotechnology).
Mouse monoclonal anti-ATP5A [15H4C4] abcam Cat#ab14748 ICC concentration: 1:100 Validated by company (abcam).
Mouse monoclonal anti-LAMP1 [H4A3] abcam Cat#ab25630 ICC concentration: 1:100 Validated by company (abcam).
Mouse monoclonal anti-Calreticulin [FMC 75] abcam Cat#ab22683 ICC concentration: 1:100 Validated by company (abcam).
Mouse polyclonal anti-GM130 abcam Cat#ab169276 ICC concentration: 1:100 Validated by company (abcam).
Mouse monoclonal anti-GFP [B-2] Santa Cruz Biotech Cat#sc-9996 WB concentration: 1:500 Validated by molecular weight in

manuscript.
Mouse monoclonal anti-Tau [HT7] Invitrogen Cat#MN1000 WB concentration: 1:1000 Validated by molecular weight in manuscript.
Sheep anti-Mouse IgG HRP-linked secondary Sigma Aldrich Cat#NXA931V WB concentration: 1:3000
Donkey anti-Rabbit IgG HRP-linked secondary Sigma Aldrich Cat#NA934V WB concentration: 1:3000
Mouse anti-Goat IgG HRP-conjugated secondary Santa Cruz Biotech Cat#sc-2354 WB concentration: 1:3000
Alexa Fluor 488 donkey anti-rabbit IgG (H+L) secondary Invitrogen Cat#A21206 ICC concentration: 1:500
Alexa Fluor 647 donkey anti-goat IgG (H+L) secondary Invitrogen Cat#A21447 ICC concentration: 1:500
Alexa Fluor 647 donkey anti-mouse IgG (H+L) secondary Invitrogen Cat#A21238 ICC concentration: 1:500

Eukaryotic cell lines

Policy information about cell lines and Sex and Gender in Research

Cell line source(s)

Fibroblasts were generated from skin biopsies, as previously described (Wang et al, 2019). Sex was not considered in the study design. Cell lines used are as follows:
F0062.1 Male human healthy skin fibroblasts provided by laboratory of Dr. Philippe Campeau, CHU Sainte Justine Research Center, Montreal, QC, Canada.
F0342.1 Male human skin fibroblasts with OXR1 mutation (c.1324delA; p.Ser44Valfs*2) provided by laboratory of Dr. Philippe Campeau, CHU Sainte Justine Research Center, Montreal, QC, Canada.

Authentication

Cell lines were authenticated at CHU Sainte-Justine, Montreal, as previously described (Wang et al, 2019). Cell lines were authenticated at sample collecting by Sanger sequencing.

Mycoplasma contamination

All cell lines tested negative for mycoplasma contamination.

Commonly misidentified lines
(See ICLAC register)

No commonly misidentified lines were used in this study.

Animals and other research organisms

Policy information about studies involving animals; ARRIVE guidelines recommended for reporting animal research, and Sex and Gender in Research

Laboratory animals

No animals relevant for reporting were used in this study. Only flies were used in this study. The analyses for this manuscript utilized longitudinal analyses across life. The strains used are listed here
Drosophila Genetic Reference Panel strains Bloomington Drosophila Stock Center All DGRP strains
Act5C-GS-Gal4 Driver (inducible, whole body)
P{w[+mC]=Act5C(FRT.y[+])GAL4.Switch.PR}X, y[1] w[*] Bloomington Drosophila Stock Center #9431
Elav-GS-Gal4 Driver (inducible, neuronal)
w1118 ; P{w[+mC]=elav-Switch.O}GSG301 Bloomington Drosophila Stock Center #43642
Elav-Gal4 Driver (non-inducible, neuronal)
P{w[+mW.hs]=GawB}elav[C155] Bloomington Drosophila Stock Center #458
5966-GS-Gal4 (inducible, intestinal)
+; 5966-GS Provided by lab of Dr. David Walker, University of California, Los Angeles
repo-Gal4 (non-inducible, glial)
w[1118]; P{w[+m*]=GAL4}repo/TM3, Sb[1] Bloomington Drosophila Stock Center #7415
GMR-driven mutant Tau (non-inducible, eye), Elav-Gal4 (non-inducible, neuronal)
P{w[+mW.hs]=GawB}elav[C155]; P{w[+mC]=GMR-htau/Ex}1.1 Bloomington Drosophila Stock Center #51360
w1118 control strain Bloomington Drosophila Stock Center #5905
Transgenic RNAi Project (TRiP) empty vector control strain
y[1] sc[*] v[1]; P{y[+t7.7] v[+t1.8]=VALIUM20-mCherry}attP2 Bloomington Drosophila Stock Center #35785
mtd RNAi
y[1] sc[*] v[1]; P{TRiP.HMS01666}attP2/TM3, Sb[1] Bloomington Drosophila Stock Center #38519
mtd mutant
y[1] w[*]; Mi{y[+mDint2]=MIC}mtd[MI02920]/TM3, Sb[1] Ser[1] Bloomington Drosophila Stock Center #76158
hOXR1 overexpression
w1118; P{UAS-OXR1.HA}1 Bloomington Drosophila Stock Center #64104
hOXR1 overexpression
w1118; P{UAS-OXR1.HA}4/TM3, Sb1 Bloomington Drosophila Stock Center #64105
Fdxh RNAi
w1118; P{GD1274}v24497 Vienna Drosophila Resource Center #24497
CG15515 RNAi
w1118; P{GD8577}v39872 Vienna Drosophila Resource Center #39872
tj RNAi
y[1] sc[*] v[1]; P{TRiP.HMS01069}attP2 Bloomington Drosophila Stock Center #34595
TJ-GFP
w[1118]; P33657Bac{y[+mDint2] w[+mC]=tj-GFP.FPTB}VK00033 Bloomington Drosophila Stock Center #66391
ctcf RNAi
y[1] sc[*] v[1]; P{TRiP.GL00266}attP2 Bloomington Drosophila Stock Center #35354
ctcf RNAi
y[1] v[1]; P{TRiP.HMS02017}attP40 Bloomington Drosophila Stock Center #40850
vps26 RNAi
y[1] v[1]; P{TRiP.HMS01769}attP40 Bloomington Drosophila Stock Center #38937
vps29 RNAi

y[1] v[1]; P{TRiP.HMJ21316}attP40 Bloomington Drosophila Stock Center #53951
vps35 RNAi
y[1] sc[*] v[1] sev2[1]; P{TRiP.HMS01858}attP40 Bloomington Drosophila Stock Center #38944
Atg8 overexpression
y[1] w[1118]; P{w[+mC]=UASp-GFP-mCherry-Atg8a}2 Bloomington Drosophila Stock Center #37749
hLAMP1 overexpression
y[1] w[*]; PBac{y[+mDint2] w[+mC]=UAS-hLAMP1.HA}VK00033 Bloomington Drosophila Stock Center #86301
Atg1 overexpression
y[1] w[*]; P{w[+mC]=UAS-Atg1.S}6A Bloomington Drosophila Stock Center #51654
mitoGFP overexpression
w[1118]; P{w[+mC]=UAS-mito-HA-GFP.AP}2/CyO Bloomington Drosophila Stock Center #8442

Wild animals

No wild animals were used in this study.

Reporting on sex

Females were primarily used due to previous research indicating that dietary restriction primarily affects female flies (Spencer et al, 2003; Magwere et al, 2004), though males were used in some studies to distinguish any sex-specific phenotypes. Sex is specified in these cases.

Field-collected samples

No field-collected samples were used.

Ethics oversight

No ethical oversight was required, as no animals relevant for ethical oversight were used in this study.

Note that full information on the approval of the study protocol must also be provided in the manuscript.
